# The Discovery of the Rare *Chara baueri* (Charales, Charophyceae) in Serbia

**DOI:** 10.3390/plants9111606

**Published:** 2020-11-19

**Authors:** Ivana Trbojević, Vanja Milovanović, Gordana Subakov Simić

**Affiliations:** Faculty of Biology, University of Belgrade, Studentski trg 16, 11000 Beograd, Serbia; b3026_2019@stud.bio.bg.ac.rs (V.M.); gsubak@bio.bg.ac.rs (G.S.S.)

**Keywords:** charophyta, *Chara baueri*, species ecology, species biogeography

## Abstract

*Chara baueri* is one of the rarest charophytes worldwide. It had been considered extinct in Europe for more than a century, from the 1870s to 2006, when it was rediscovered in Germany. The current distribution of this species is limited to a few localities in Europe (Germany, Poland and Russia), and one locality in Asia (Kazakhstan). We present a new finding of *Chara baueri*, to be a significant contribution to the species ecology and biogeography, and helping to review and update the current scarce knowledge. *Chara baueri* was discovered in Serbia and monitored for two vegetative seasons in 2018 and 2019, along with the associated macrophyte vegetation and water quality parameters. The morphology and ecology data of the species are presented comparatively with the literature data and the biogeography is critically reviewed. The population in Serbia is the first verified record of *Chara baueri* in southern Europe. Considering the recent findings and the knowledge accumulated in these records, *Chara baueri* was very possibly never extinct at all, but overlooked in Europe for the entire 20th century. We suggest that waterfowl migrating from the northern parts of Europe should be considered as the important spreading agent of *Chara baueri* in southern regions.

## 1. Introduction

*Chara baueri* A. Braun is considered to be one of the rarest charophytes worldwide [1]. It was first discovered and often collected in the Berlin area in the first half of the 19th century, until the 1870s. During the 19th century, only a few other localities all over the world could be reliably recognized–in Austria, southern Sweden and near Schwerin in Germany [2], all of them single records from the first half of the 19th century. Since the end of the 19th century, *C. baueri* was thought to be extinct in Europe [2,3]. The first record of *C. baueri* since then was made far away from the formerly known localities, in Kazakhstan in 1994, and this is still the only record in Asia [4]. Recently, it was rediscovered in Germany (Brandenburg) in 2006 [2] and newly discovered for Poland (2008) [5,6] and Russia (2010) [7].

*C. baueri* is morphologically, on first sight, very similar to *Chara braunii*, which is commonly found all over the world [3,8]. Still, there is a clear difference between these two taxa: a triplostique cortex that develops on the main axis (at least at the upper internodes) of *C. baueri*, and well-developed solitary spines, while *Chara braunii* is completely ecorticated [2,3,6]. Although Krause [3] suggested that these two species could be closely related, this has not yet been proven by molecular phylogenetic analysis. However, it has been confirmed that *C. baueri* could be even more phylogenetically close to the genus *Lamprothamnium* than to the other *Chara* species [9]. Phylogenetic relationships of these rare charophyte remain to be resolved in future studies, and collecting material from a wide geographic range is crucial so that these studies could offer a reliable answer.

The purpose of this study is to report about the first record of *C. baueri* in Serbia (ponds in the locality Štrbac, in the special nature reserve (SNR) “Gornje Podunavlje”). Discovered populations were monitored for two vegetative seasons in 2018 and 2019, along with the associated macrophyte vegetation and water quality parameters. The specimens of *C. baueri* are morphologically described in detail, comparative with the reliable literature sources. Habitat characteristics are discussed in terms of updating knowledge on the ecology of this rare charophyte. This new finding of *C. baueri* is discussed as a significant contribution to the knowledge on this species biogeography.

## 2. Results

The measured environmental parameters in pond 1 and pond 2 are presented in Table 1. It can be noticed that values of parameters are quite variable, since these are small and very shallow ecosystems. The measured values for total phosphorus (TP) and total nitrogen (TN) point to both ponds being eutrophic water bodies. The other measured parameters were more or less in range of moderate values. *C. baueri* was found in pond 1 in 2018 in July and August, and in 2019, at the end of July (31st), young plants were detected. In pond 2, *C. baueri* was found in August and September 2018. The bottoms of the both ponds were covered with a thick layer of very fine silt.

The following macrophyte and Charophyte species, apart from *C. baueri*, were recorded at the sampling sites: *Eleocharis palustris* agg., *Hydrocharis morsus-ranae* L., *Myriophyllum spicatum* L. (dominant in Pond 1), *Potamogeton gramineus* L., *Potamogeton nodosus* Poir., *Ranunculus aquatilis* agg., *Salvinia natans* (L.) All., *Schoenoplectus lacustris* (L.) Palla, *Spirodela polyrhiza* (L.) Schleid., *Utricularia* L. sp., *Chara braunii* C.C.Gmel., *Chara globularis* Thuill., *Chara tenuispina* A.Braun, *Nitella capillaris* (Krock.) J.Groves & Bull.-Webst., *Nitella mucronata* (A.Braun) Miq. and *Nitella* C.Agardh sp. In general, macrophyte vegetation was better developed (in terms of both diversity and cover) in pond 1 (more or less stable water level) in comparison to pond 2 (more prone to drastic water level changes). When pond 2 was filled with water, usually flotant macrophyte species dominated. In pond 1, *Myriophillum spicatum* was found to be dominant in the deeper parts along with *Potamogeton nodosus*, while the shallower part was completely covered with a meadow of *Chara globularis* and *Chara tenuispina*, and in the most shallow region, *C. baueri* formed patchy groups (*Nitella* specimens were found only sporadically, only a few specimens in total). Although we expected ponds to dry up completely at some point during the summer period, and despite the very dry August and September 2018, that did not happen.

### Description of C. Baueri from Serbia

**Figure 1:**
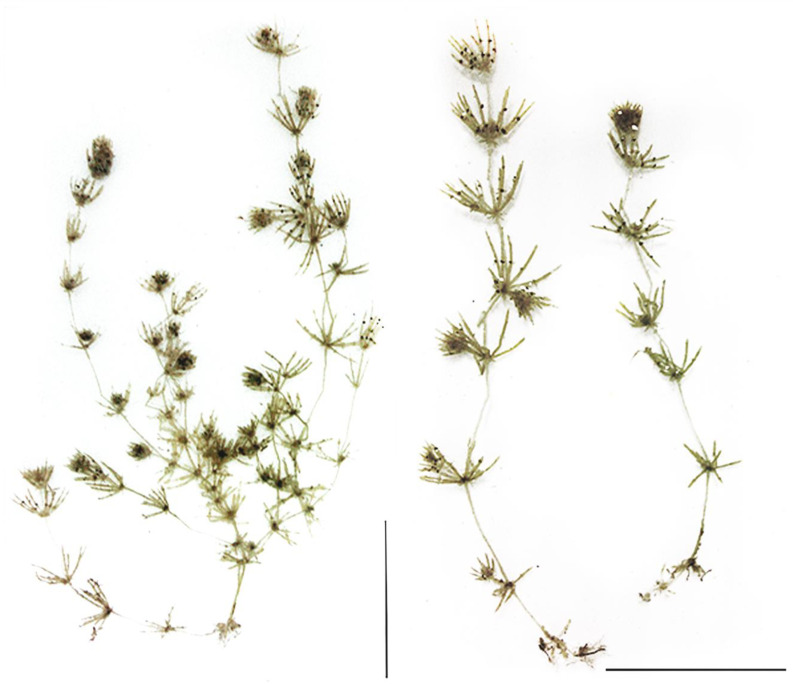
Macro habitus, scale 5 cm.

**Figure 2:**
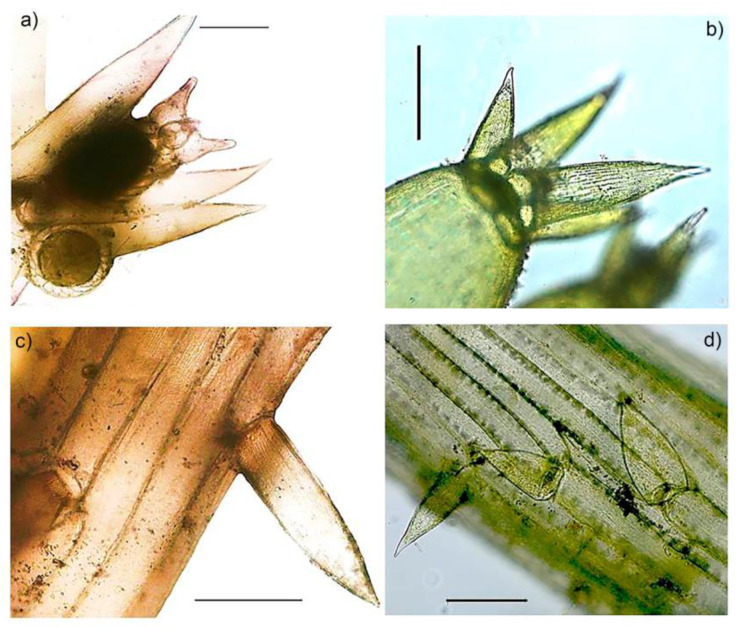
Microscopic taxonomic features: (**a**) Gametangia, (**b**) terminal corona on the branchlet terminal segment, (**c**) triplostichous, isostichous cortex, (**d**) cortex detail showing irregular structure, scale 200 µm.

The macro habitus of the discovered specimens of *C. baueri* are presented in Figure 1. Specimens were found in groups in very shallow water (0.1 to 0.3 m), almost on the shoreline, in areas prone to drying up due to water level changes. Triplostichous and isostichous cortex on axis was clearly noticeable throughout the stem, though in some parts irregular (Figure 2c,d). This is an important taxonomic parameter for distinguishing this species from *C. braunii*. Specimens were fructifying, with both female and male gametangia being well developed (Figure 2a,b). Ripe oospores were also abundantly represented. Oospores were large and markedly black, while the oospore membrane color was light brown and showed a finely granulated structure.

Specimens of *C. baueri* found in Serbia are described in detail, and presented comparatively with the data from the relevant literature sources [1,4,6,10,11,12,13,14,15] (Table 2).

## 3. Discussion

**Figure 3:**
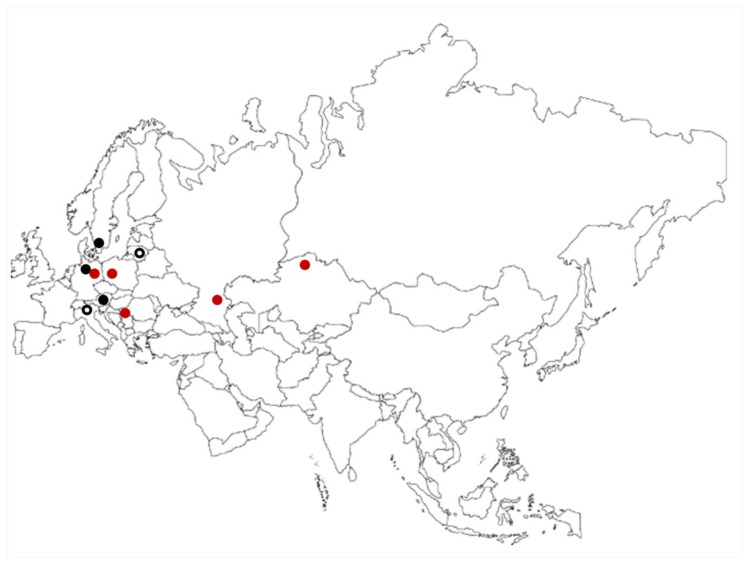
Distribution of *C. baueri* across Eurasian continent: Red circles—recent findings, new discovery or rediscovery. Black circles—reliable historical findings. Empty black circles—historical records which could not be confirmed or verified.

In the context of biogeography of the species, it is very interesting to review the historical and current distribution of *C. baueri*. Recent records are known from central, southern and eastern Europe, and central Asia (Figure 3). Fully reliable historical findings are known only from central/northern Europe (Germany and Sweden [2]). Other historical records (in Italy [16] and Lithuania [17]) could not be confirmed or verified [2]. As far as the authors’ knowledge reaches, the historical finding from Austria [13] also cannot be supported by the herbarium material, but according to Krause [3], Raabe [2] and other authors’ opinions, it should be considered valid (Figure 3).

Considering the description of his own material collected in the ponds near St. Andrä in southern Austria, and the drawing of these specimens, Ganterer’s [13] finding is likely reliable. The most interesting point is that this locality in southern Austria is relatively close to the locality in Serbia, only about 340 km in a straight line [18]. While comparing his specimens with Bauer’s original material, in which only a few upper internodes or even only one internode was corticated, Ganterer [13] commented that the plants he found near St. Andrä were larger and corticated throughout the stem, with long and numerous spine cells. This peculiarity was also seen in the specimens found in Serbia, which were large, and the cortex was present throughout the stems, with long spine cells (Table 2). Habitat specificities, as well as population origin and/or isolation, could be the basis of these specificities. Still, after Ganterer’s record, *C. baueri* was never confirmed again in Austria, or anywhere near. The record from Serbia that we are reporting here is the only verified one in southern Europe. The fact is that recent findings (Figure 3) substantially widen the distribution area of *Chara baueri* in comparison to the historical records. In Germany, *C. baueri* is considered highly endangered (“stark gefährdet”), it is threatened and under the risk of extinction in Poland, and it is regionally extinct in Sweden [19].

Data on the ecology of the *C. baueri* are very scarce, and authors could only reach data published by Pukacz et al. [1] and Doege et al. [20] (since data presented in Doege et al. [20] are practically the same as in Pukacz et al. [1], we are further referencing the older, but original publication). When the literature data is compared to the data obtained in this study (Table 3), it is clear that the values for almost all comparable parameters are lower, meaning that water in the localities in Serbia were poorer in Ca and Mg content and electrolytes in general (conductivity), as well as softer and a bit more alkaline (Table 3). Considering the nutrient content, lower concentrations were detected in our localities, though according to the total phosphorus (TP) and total nitrogen (TN), both ponds in Serbia were also eutrophic water bodies, as expected [21].

Recently added details on the habitat type of the species *C. baueri* [21] correspond to the characteristics of the localities in Serbia—small water bodies in the fields, rich in nutrients; in Serbia, those are ponds, artificially made to serve as a watering place for wild animals, prevalently wild boars and deer (locality Štrbac, where ponds are placed in the special nature reserve (SNR) “Gornje Podunavlje”). Digging the watering places in the fields of Štrbac in the SNR “Gornje Podunavlje”, could have activated a diaspore bank with *C. baueri* oospores in it, which could explain the surprising occurrence of this rare charophyte in this area. Sediment characteristics and the depth range where the species occurs matches literature [6,21] descriptions.

Among the most often associated macrophyte species that Gregor [21] emphasized, in our study, only *Nitella mucronata* and *Chara globularis* were recorded along with *C. baueri*. Nevertheless, *Elatine alsinastrum* which was recorded in almost all literature sources as being associated with *C. baueri* [21], was not detected in the localities in Serbia. Considering the phenology of the *C. baueri* species, it is clear that its yearly occurrence depends on the existence of ephemere habitats where it typically grows [21].

When considering the taxonomically relevant morphological characteristics of *C. baueri*, the triplostichous and isostichouse cortication is a distinctive feature, clearly separating this taxon from *C. braunii*, which is completely ecorticated [21] (see Table 2). Still, Langangen and Sviridenko [4] ascertained that the specimens found in Kazakhstan had a diplo- to triplo-stichous cortex, while young internodes were mostly diplostichous. These authors also described the old Swedish specimen from 1849 as mostly 2 corticate, although they say it was difficult to determine, probably because the material was old and the herbarium specimen was studied in dry conditions [4]. Urbaniak and Gąbka [6] remarked the irregularity of the cortex, which was also noted in this study. It was already shown that the number of cortex cell rows may be variable within a genetically homogeneous *Chara* group [22], which may be the case in *C. baueri* as well.

Finally, the origin of the population of *C. baueri* in Serbia is debatable. Langangen and Sviridenko [4] suggested that long-distance dispersal by birds migrating from Europe to the Kazakhsthan area in the spring could explain the occurrence of *C. baueri* in Central Asia. We find this hypothesis plausible, as it could potentially explain the finding in Serbia. The SNR “Gornje Podunavlje”, where the locality Štrbac and studied ponds belong, is declared a Ramsar site and Important Bird Area (IBA), where many migratory bird species stop to rest or nest, and this area is the most significant national nesting area of the wild (graylag) goose (*Anser anser*) in Serbia [23]. For the sake of illustration, the distribution range of this waterfowl species covers all recent localities of *C. baueri* (in Europe and Asia) [24]. Also, according to Dick et al. [25], the central European graylag goose population migration routes perfectly link all localities where *C. baueri* was ever found in Europe (including non-reliable findings). Considering the habitat characteristics of *C. baueri*, various waterfowl species migrating from the north of Europe could be dispersal agents in more southern regions, and IBA and nesting areas of these birds should be the first ones surveyed in search for the potential new localities of *C. baueri* in Europe. Also, we suggest that future detailed multidisciplinary research on if and how the migration routes of waterfowl coincide with the distribution of other charophytes across Europe would be valuable input regarding charophyte biogeography, especially rare species.

Summarizing the current knowledge of ecological requirements and habitat characteristics of *C. baueri*, as well as the distribution range, it is quite possible that this species was simply overlooked in Europe for the entire 20th century. Contributing to this knowledge gap is the lack of organized, targeted and continued monitoring of charophytes (i.e., certain type of habitats), which are often completely overlooked in macrophyte studies or at best recognized as *Chara* sp.

## 4. Materials and Methods

**Figure 4:**
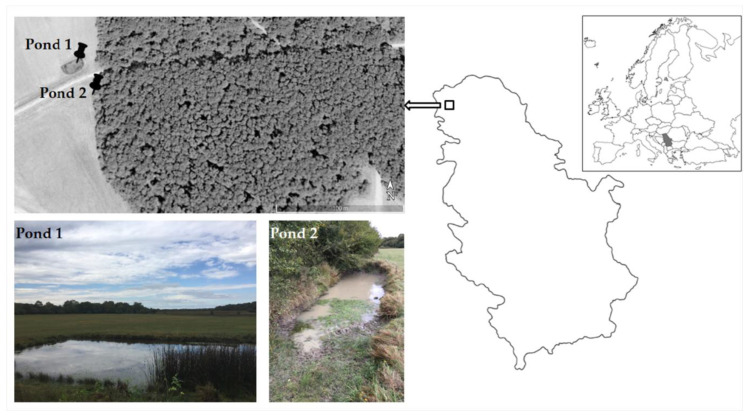
Study area and sampling localities.

The study area was located in the Special Nature Reserve (SNR) “Gornje Podunavlje”, Štrbac area, in Serbia’s Northern Province, Vojvodina (Figure 4). This area is a very unique and complex mosaic of meadows, woods, ponds and wetlands, and it includes the river Danube and its meanders.

Two ponds separated by a dirt road, located in the meadow near the woods (Figure 4), were labeled as pond 1 (N 45.812775, E 18.960583) and pond 2 (N 45.812577, E 18.960785). They were monitored monthly from May until September in 2018 and May until July in 2019. According to the nature reserve rangers, these ponds were man-made with the purpose of forming a watering place for wild animals.

Each time the ponds were sampled for charophytes, the environmental parameters: temperature (T), pH, oxygen concentration (O_2_ mg/L) and saturation (O_2_%), and conductivity, were measured in situ in the ponds, using digital field instruments made by Eutech Instruments Oakton^®^ and YSI ProODDO Optical Dissolved Oxygen Meter. Simultaneously, water samples from both ponds were also taken for further laboratory analyses of chemical water properties, which were conducted in the accredited laboratories of the Institute of Public Health of Serbia “dr Milan Jovanović Batut”, using standard analytical methods.

Charophytes were collected by wading and using rakes and grapnels. Material was stored in plastic bags and transported to the laboratory where it was identified using a STEMI DV4 stereomicroscope and a Nikon YS100 microscope and standard literature [3,6,14,16,17,26].

Micrographs were made using a Carl Zeiss AxioImager M1 microscope and a digital camera AxioCam MRc5, with AxioVision 4.8 software. Part of the identified material was herbarized and part was stored in 4% formalin (final concentration) in the collection of wet specimens of the Department of Algology, Mycology and Lichenology, Faculty of Biology, University of Belgrade (BEOU, Belgrade, Serbia).

## 5. Conclusions

This study reported the first record of *C. baueri* in Serbia, but also the first reliable record of the species in southern Europe. Results of our study supplement the knowledge on the habitat characteristics and overall ecology of this rare charophyte. Our finding significantly contributes to the species biogeography, which is reviewed and discussed, thus concluding that the distribution of *C. baueri* should be observed across the Eurasian continent. Waterfowl species migrating from the north of Europe are suggested as the most probable dispersal agent of *C. baueri* in more southern regions, where IBA and nesting areas of these birds could be considered the potential new localities of *C. baueri* in Europe. Considering recent findings and knowledge accumulated in these records, *C. baueri* has very possibly never been extinct, but overlooked in Europe for the entire 20th century.

## Figures and Tables

**Table 1:** Environmental parameters measured in pond 1 and pond 2 in 2018 and 2019. Bold is for the dates when *Chara baueri* was found in ponds. Water temperature (T), oxygen concentration/saturation (O_2_), pH, conductivity (Cond.), degree of General Hardness of water (°dH), calcium ions concentration (Ca^2+^), magnesium ions concentration (Mg^2+^), nitrites (NO_2_^−^), nitrates (NO_3_^−^), ammonia (NH_4_^+^), total nitrogen (TN), Orthophosphates (OP), total phosphates (TP).

	T	O_2_	O_2_	pH	Cond	°dH	Ca^2+^	Mg^2+^	NO_2_^-^	NO_3_^-^	NH_4_^+^	TN	OP	TP
	°C	mg/L	%		µS/cm		mg/L	mg/L	mg/L	mg/L	mg/L	mg/L	mg/L	mg/L
**POND 1**														
May 2018	20.5	9.05	102	8.23	325	**-**	**-**	**-**	**-**	**-**	**-**	**-**	**-**	**-**
June 2018	22	4.42	50.5	8	300	**-**	**-**	**-**	**-**	**-**	**-**	**-**	**-**	**-**
**July 2018**	**30.7**	**15.45**	**190**	**9.35**	**338**	**1.1**	**3.7**	**2.7**	**0.021**	**<0.5**	**0.24**	**2.5**	**<0.020**	**0.028**
**August 2018**	**31**	**12**	**162**	**8.76**	**379**	**2.6**	**8.8**	**5.7**	**<0.020**	**<0.5**	**0.23**	**1.9**	**<0.020**	**0.026**
September 2018	14.5	9.94	101.2	8.68	346	6.3	30.2	9.1	0.027	<0.5	<0.05	2.9	<0.020	0.031
May 2019	18	15	152.6	9.61	328	5.2	12	15	<0.005	<0.5	2.2	1.2	<0.010	0.24
June 2019	29.15	11.9	157.5	9.13	344	3.7	11.6	9	<0.005	<0.5	0.45	1.5	<0.010	0.07
**July 2019**	**28.3**	**2.57**	**32.4**	**8.13**	**445**	**6.1**	**23.2**	**12.4**	**<0.005**	**<0.5**	**0.54**	**1.9**	**<0.010**	**0.07**
**POND 2**														
May 2018	**-**	**-**	**-**	**-**	**-**	**-**	**-**	**-**	**-**	**-**	**-**	**-**	**-**	**-**
June 2018	21	2.21	25.4	7.56	275	**-**	**-**	**-**	**-**	**-**	**-**	**-**	**-**	**-**
July 2018	28.1	7.5	96.5	8.12	342	6.1	31.2	7.5	0.043	<0.5	0.21	1.7	<0.020	0.029
**August 2018**	**26.1**	**6.24**	**77.1**	**8.44**	**435**	**4.4**	**21.8**	**5.9**	**<0.020**	**<0.5**	**0.18**	**3**	**<0.020**	**0.046**
**September 2018**	**15**	**12**	**120**	**9.08**	**355**	**6.7**	**33.8**	**8.4**	**0.08**	**0.91**	**0.79**	**5.3**	**<0.020**	**0.04**
May 2019	15.5	13.7	135.2	9.18	352	**-**	**-**	**-**	**-**	**-**	**-**	**-**	**-**	**-**
June 2019	28	3.63	43.6	7.96	354	**-**	**-**	**-**	**-**	**-**	**-**	**-**	**-**	**-**
July 2019	25.6	2.42	28.4	8.13	416	**-**	**-**	**-**	**-**	**-**	**-**	**-**	**-**	**-**

**Table 2:** Species traits of *Chara baueri*, as described in the literature, and traits measured in samples of *C. baueri* discovered in Serbia (19 specimens and 127 oospores were measured for obtaining presented values, measured material was previously conserved in formalin).

Literature Source	Reichenbach, 1829 [10]	Braun, 1835 [11] Braun and Nordstedt, 1882 [12]	Ganterer, 1847 [13]	Migula, 1897 [14]	Langangen and Sviridenko, 1995 [4]	Hutorowicz, 2007 [15]	Pukacz, 2012 [1]	Urbaniak and Gąbka, 2014 [6]	Specimens from Serbia
**Note**					Specimen from Kazakhstan (1994)/ Specimen from Sweden (1849), herbarium specimen examined in dry condition	Oospores from specimens from Kazakhstan [4]	Oospores from specimens from Cedynia, Western Poland (August 2008)/ Oospores from specimens from near Batzlow, Germany (2006)		
**Habitus description**	Nice green color, turning black towards the bottom. Stiff stature, almost cartilaginous, ‘’shining’’ similarly to *Ceratophyllum*.	Hard to differentiate from *Ch. coronata* (*Chara braunii*) without the loupe	Richly branched, bushy habitus, mostly light green in color	Habitus is not easy to distinguish from *Ch. coronata* (*Chara braunii*), all parts are proportionally thicker. Light yellow-green or green, rarely brownish-green.	**-**/**-**	**-**	**-**	Rather small plants, richly branched, yellow green to light green, strongly resembling *C.braunii*	Light green to yellowish green. Resembling *C. braunii*, but branchlets more rounded, plump and voluminous—like succulent.
**Axes**									
1. Incrustation	**-**	Not incrusted	**-**	Incrustation is abundant	Not incrusted/ slightly incrusted	**-**	**-**	Slightly incrusted	Very slightly incrusted
2. Diameter			More than 1.0975 mm (more than ½ Linie, 1 Linie = 2.195 mm)	Up to 1 mm	Up to 0.57 (0.65 in Figure 2 caption) mm/0.93 mm			0.6–2.1 mm	0.55–0.95 mm
**Cortex**	**-**	Finely striped cortex	In the uppermost internodes, or throughout the stem more or less clearly and finely striped, and covered with scattered spines	Triplostichous, isostichous	2–3 corticate, isostichous, young internodes mostly 2 corticate/ Mostly 2 corticate, isostichous (hard to determine in dry material)	**-**	**-**	Triplostichous, in some parts irregular	Triplostichous, isostichous cortex developed throughout the stem, in some parts irregular
**Spine cells**									
1. Description	**-**	Fine, spiked	**-**	Solitary, up to ½ of axes diameter, acuminate–spiky, especially in young segments	Solitary, acute, to 1 x stem diameter, commonly shorter, many on young internodes/ Solitary, acute, many in young internodes, only few in the lower parts of stem	**-**	**-**	Solitary, common, acuminate, not exceeding axes diameter	Large, solitary, acuminate, more numerous in the upper section of axes
**Branchlets**									
1. Number in a whorl and description	**-**	Ecorticated	Ecorticated	8–9	8–9, ecorticated/ 7–8, ecorticated	**-**	**-**	7	8–11, ecorticated
**Oogonia**									
1. Number in node	Usually geminate, rarely 1 or 3	**-**	Single, geminate or 3, ovoid	**-**	**-**	**-**	**-**	**-**	1, mostly 2, sometimes even 3
2. Dimensions (length × width)	**-**	700–880 µm × 380–480 µm	**-**	650 × 500 µm	1000 µm/ 600 µm × 250 µm (immature)	**-**	**-**	585–950 µm × 365–610 µm	580–710 (775) µm × 370–450 µm
3. Number of convolutions	8–10	10–11	Mostly 10	8–10	**-**	**-**	**-**	**-**	9–10
4. Coronula width × height	**-**	140–200 µm × 220–250 µm		150–200 µm	**-**	**-**	**-**	**-**	(120) 170–260 (275) µm × (150) 200–330 (350) µm
**Oospore**									
1. Color	**-**	**-**	**-**	Black	**-**/Black	Light brown to black/**-**	Dark brown or black/**-**	Black/dark brown	Black
2. Dimensions (length × width)	**-**	500–560 µm × 310–350 µm	**-**	500–550 µm × 280–340 µm	**-**/600 µm	436–574 µm × 281–340 µm	400–667 µm × 183–300 µm/417–550 µm × 216–300 µm	465–740 µm × 280–340 µm	475–620 µm × 230–350 µm
3. Ridges description	**-**	**-**	**-**	Irregular, blunt/sharp edges	**-**	Unpronounced in young and prominent in mature oospores	Prominent ridges/**-**	**-**	Oval, blunt
4. Ridges number	**-**	**-**	**-**	8	**-**	8–11	8–11 (most often 9)/8–11	**-**	8–10 (11)
5. Fossa	**-**	**-**	**-**	**-**	**-**	**-**	**-**	**-**	50–70 µm
6. Membrane coloration	**-**	**-**	**-**	**-**	**-**	**-**	**-**	**-**	Light brown
7. Membrane structure	**-**	**-**	**-**	**-**	**-**	Either smooth or finely granulated	**-**	**-**	Finely granulated
**Antheridia**									
Diameter and description	In pairs or individual, brick red color	280–370 µm	Individual or in pairs, beneath the oogonia	250–300 µm	**-**/200 µm	**-**	**-**	250–330 µm, below oogonia	250–300 µm, below oogonia, individual, brick red color

**Table 3:** Summed available data on the ecology of *C. baueri* according to Pukacz et al. [1], comparatively with the data obtained in this study (mean values presented).

	O_2_ (mg/L)	Hardness (°dH)	pH	Conductivity (µS/cm)	Mg^2+^ (mg/L)	Ca^2+^ (mg/L)	TP (mg/L)	PO_4_^−^ (mg/L)	TN (mg/L)	NO_2_^−^ (mg/L)	NO_3_^−^ (mg/L)	NH_4_^+^ (mg/L)
**Pukacz et al., 2012 [1]**												
Cediniya (Poland)	6.14	12.9	8.01	611	14.9	71.7	1.04	0.63	3.89	**-**	0.47	1.31
Batzlow (Germany)	3.25	13.2	7.92	632	16.3	67.6	1.12	0.71	5.15	0.02	0.68	1.45
**This study**												
Pond 1	10.04	4.17	8.7	350.6	8.98	14.92	0.08	<0.015	1.98	0.02	<0.5	0.73
Pond 2	6.8	5.73	8.3	361.3	7.27	28.93	0.04	<0.02	3.33	0.06	0.91	0.39
